# From innate-like to innate: the next wave of off-the-shelf CAR immunotherapies

**DOI:** 10.3389/fimmu.2025.1691743

**Published:** 2026-01-09

**Authors:** Ying Feng, Zhibo Yang, Yueru Zhou, Ying Liang, Hai Zhao

**Affiliations:** 1 Department of Emergency, The Affiliated Hospital of Qingdao University, Qingdao, Shandong, China; 2 Department of Neurosurgery, 3201 Hospital of Xi’an Jiaotong University Health Science Center, Hanzhong, Shaanxi, China; 3 Department of Outpatient, The Affiliated Hospital of Qingdao University, Qingdao, Shandong, China; 4 Department of Neurosurgery, The Affiliated Hospital of Qingdao University, Qingdao, Shandong, China

**Keywords:** Keywords: allogeneic, CAR-iNKT, CAR-macrophage, CAR-NK, CAR-γδ T, IL-15 armoring, IPSC, MAIT

## Abstract

While autologous CAR T-cell therapies have revolutionized the treatment of hematologic malignancies, their widespread application is hindered by manufacturing complexities, high costs, and limited efficacy against solid tumors due to antigen heterogeneity and the TME. Moreover, the logistical burden of bespoke patient-specific manufacturing restricts global scalability. In response, the immunotherapy landscape is pivoting toward “off-the-shelf” allogeneic therapies derived from innate and innate-like effectors. This review provides a comprehensive analysis of four emerging platforms: CAR-NK cells, CAR-NKT cells, γδ T cells, and CAR-M. Unlike conventional αβ T cells, these lineages utilize MHC-independent mechanisms to recognize stress-induced ligands or lipid antigens, inherently minimizing the risk of GvHD while enabling standardized, batched manufacturing. We critically examine the diverse manufacturing paradigms, contrasting the scalability of iPSC-derived sources with the accessibility of umbilical cord blood products. Furthermore, we detail advanced engineering strategies designed to overcome the lineage-specific limitations revealed by early trials—specifically, “armoring” constructs with IL-15 to boost *in vivo* persistence and metabolic reprogramming to sustain function within the TME. Finally, we synthesize emerging clinical evidence which confirms the favorable safety profile of these allogeneic approaches but highlights persistent bottlenecks: limited durability of response, cryopreservation-induced loss of viability, and batch-to-batch variability. We conclude that unlocking the full potential of innate CAR therapies requires a dual focus on harmonizing manufacturing controls and developing next-generation engineering logic to ensure durable control of solid tumors.

## Introduction

Autologous chimeric antigen receptor (CAR) T-cell therapy has produced durable remissions in hematologic malignancies; however, its broad application is constrained by the high cost and logistical burden of bespoke manufacturing (1–4). Furthermore, in solid tumors, efficacy is severely limited by antigen heterogeneity, physical stromal barriers, and a profoundly immunosuppressive tumor microenvironment (TME) (5–7). While extensive engineering efforts—ranging from cytokine “armoring” (e.g., IL-15, IL-18) and logic-gated circuits to CRISPR-mediated checkpoint disruption—have been deployed to enhance CAR-T potency and persistence, these iterative refinements do not address the fundamental limitations of the conventional αβ T-cell scaffold: the reliance on autologous cells and the intrinsic risk of alloreactivity (graft-versus-host disease) in allogeneic settings (8–12). Given these persistent challenges, attention has shifted to immune lineages whose native biology may complement or surpass conventional T cells. Innate and innate-like lymphocytes—natural killer (NK) cells, invariant natural killer T (NKT) cells, and γδ T cells—provide rapid stress surveillance and MHC-independent, donor-unrestricted target recognition (13–16). These properties inherently reduce the risk of graft-versus-host disease, paving the way for standardized, “off-the-shelf” allogeneic products that can be manufactured at scale and administered on demand. In parallel, macrophages offer a distinct advantage within the solid tumor niche; engineering these professional phagocytes with CARs seeks not only to devour malignant cells but also to reprogram the myeloid-rich TME, potentially bridging innate and adaptive immunity. This Review synthesizes the rationale, design principles, and emerging clinical evidence for these next-generation platforms. We critically compare their biological advantages with those of CAR T cells; outline engineering levers—such as metabolic rewiring and trafficking upgrades—that tune function; and examine manufacturing routes compatible with universal deployment. Finally, we highlight the translational gaps that must be bridged—particularly regarding *in vivo* durability and cryopreservation—to unlock their full potential against solid tumors.

## CAR NK cells

Human natural killer (NK) cells are innate lymphoid effectors defined phenotypically by CD56 and CD16 expression—classically partitioned into a cytokine-oriented CD56^bright^ CD16^lo^ subset and a predominantly cytotoxic CD56^dim^ CD16^bright^ subset—and by the absence of the αβ T-cell receptor (TCR) (17, 18). Equipped to recognize and eliminate malignant targets without reliance on major histocompatibility complex class I or II, human NK cells engage “stress” signals via the C-type lectin–like activating receptor NKG2D and its ligands on transformed cells. Upon target encounter, they deploy a multifaceted cytotoxic program that integrates perforin–granzyme release and inflammatory cytokine secretion with death-receptor pathways (for example, FasL and TRAIL) and antibody-dependent cellular cytotoxicity mediated through FcγRIIIa (CD16) (19).

Because such potent cytotoxicity must be constrained to protect healthy tissues, NK cell activity is continuously “tuned” by constitutively expressed inhibitory receptors—including killer cell immunoglobulin-like receptors and the CD94–NKG2A heterodimer—whose ligands (notably MHC-I) are broadly displayed by normal cells (20, 21). This inhibitory surveillance underlies the canonical “missing-self” response, whereby cells that lack or downregulate MHC-I—such as many virally infected or tumor cells—are preferentially targeted (22). Crucially, the lack of an αβ TCR means NK cells are not inherently alloreactive and do not precipitate graft-versus-host disease (GvHD) in normal tissues (23). These features, together with their innate tumor-directed cytolytic capacity, motivated clinical exploration of adoptive transfer using ex vivo–expanded allogeneic NK cells, yielding encouraging activity in hematologic malignancies such as acute myeloid leukemia (AML) (24, 25). This body of experience has, in turn, provided a practical springboard for developing chimeric antigen receptor–engineered NK (CAR-NK) products—off-the-shelf, antigen-specific cellular therapies designed to couple robust antitumor efficacy with a low risk of GvHD.

### Manufacturing of CAR NK cells

Compared with the relatively uniform, autologous CAR T-cell workflow—peripheral blood as starting material, standardized activation/transduction/expansion steps, predefined release testing for identity, purity, sterility and potency, followed by cryopreservation and on-demand administration—the allogeneic CAR NK manufacturing landscape is markedly heterogeneous. Programs differ at the very first step (donor source), drawing on peripheral blood or umbilical cord blood (UCB), and diverge further in how cells are enriched, activated, and expanded (26). In practice, centers deploy distinct cytokine cocktails (for example, IL-2/IL-15 with or without IL-21) and/or various engineered feeder systems that present costimulatory ligands and membrane-bound cytokines to drive proliferation and maturation, yielding products with comparable *in-vitro* functionality but nontrivial differences in composition and phenotype (27, 28).

This diversity complicates head-to-head comparisons, hinders the definition of universal release criteria, and poses challenges for scale-up and technology transfer. Even when manufacturing outputs meet local specifications, cross-site reproducibility and batch-to-batch consistency remain key barriers to widespread clinical deployment. A further unresolved issue is whether allogeneic CAR NK products can be cryopreserved with reliably preserved post-thaw viability, phenotype, and cytotoxic potency; many early clinical experiences have favored fresh, non-cryopreserved infusions, which improves biological performance but imposes narrow scheduling windows and substantial operational complexity for treatment centers (27, 28).

To converge on more standardized, bankable products, two complementary routes are being advanced: induced pluripotent stem cell (iPSC)–derived NK cells, which enable clonal master banks, precise genetic engineering, and large-scale, lot-controlled manufacturing; and NK cell lines, which offer highly reproducible expansion and straightforward engineering for rapid prototyping and potential off-the-shelf use (14, 29, 30). Together, these efforts aim to reduce inter-product variability, enable rigorous quality control, and support a distribution model closer to that of conventional biologics—without sacrificing the lineage-specific advantages that motivate CAR NK development in the first place.

A pivotal challenge restricting the standardization of allogeneic CAR-NK therapies lies in the intrinsic heterogeneity introduced by donor sources and activation protocols (31). While umbilical cord blood (UCB) offers a rapidly available, off-the-shelf source with reduced HLA-matching requirements, UCB units vary significantly in nucleated cell count and NK progenitor frequency, complicating batch-to-batch consistency (32). Furthermore, the choice of expansion platform creates a functional dichotomy: engineered feeder cells typically drive superior fold-expansion and cytotoxic potential compared to feeder-free cytokine cocktails, yet they introduce phenotypic variability and regulatory complexities regarding feeder clearance (33).

Clinically, this heterogeneity manifests as variable persistence. Unlike CAR-T cells, which can establish long-term memory pools, adoptively transferred NK cells often succumb to rapid exhaustion due to the lack of autocrine IL-2 support and the immunosuppressive TME (34). Early clinical data underscore that without engineered cytokine support; allogeneic NK cells may persist for only weeks. This biological limitation has cemented the inclusion of ectopic IL-15 (or IL-15/IL-15Rα fusion) as a non-negotiable ‘armoring’ feature in next-generation designs, essential for bridging the gap between transient effector function and durable disease control.

### Preclinical studies with CAR NK cells

Off-the-shelf, allogeneic CAR-NK products are now being actively investigated across hematologic malignancies. Acute myeloid leukemia (AML) is particularly susceptible to NK-cell cytotoxicity, providing a strong biological rationale to retarget NK cells against myeloid-associated antigens and thereby amplify intrinsic antileukemic activity; accordingly, CAR-NK constructs specific for CD33 and CD123 have shown robust activity in preclinical systems (35–37). In B-cell cancers, CAR-NK cells derived from diverse sources—including the NK-92 line and induced pluripotent stem cells (iPSCs)—have demonstrated antitumor efficacy when directed at CD19, CD20, or CD70 (29, 38, 39). In multiple myeloma, targeting CD138 or B-cell maturation antigen (BCMA) has yielded antimyeloma effects in xenograft models (40, 41). Notably, CAR-NK cells offer distinct advantages for T-cell malignancies: autologous CAR-T approaches aimed at pan-T markers (for example, CD5) risk inadvertent transduction of circulating malignant T cells, whereas allogeneic CAR-NK cells avert this hazard and, because NK cells do not express CD5, are intrinsically resistant to fratricide and the manufacturing-related exhaustion observed with T-cell products (42, 43). Beyond blood cancers, allogeneic NK cells are being engineered against solid-tumor antigens previously validated in CAR-T programs—such as EGFRvIII, HER2, GD2, and B7-H3—expanding their potential applicability to solid tumors as well (44–48).

**Figure 1:**
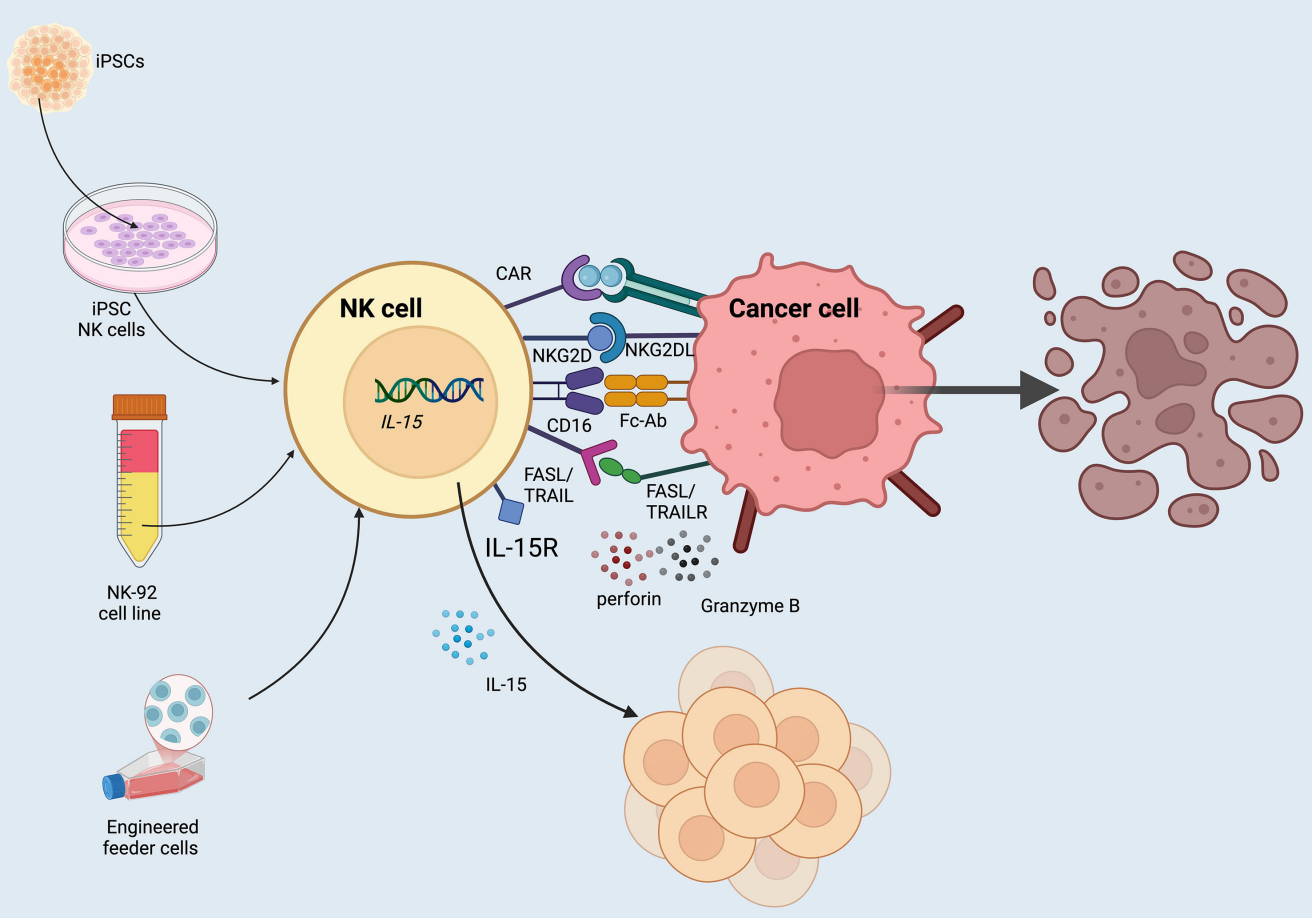
CAR-redirected NK cells. Primary human NK cells can be isolated from peripheral blood or umbilical cord blood (UCB), followed by selection and large-scale expansion with the aid of engineered feeder cells designed to promote NK cell proliferation. Alternatively, CAR NK cells can be derived from induced pluripotent stem cells (iPSCs) or established NK cell lines such as NK-92. Because NK-92–derived CAR NK products retain tumorigenic potential, they must undergo irradiation prior to clinical infusion to ensure safety. Moreover, incorporation of interleukin-15 (IL-15) into CAR constructs plays a pivotal role in enhancing the *in vivo* persistence, expansion, and functional activity of CAR NK cells.

Across immunodeficient mouse models, human CAR-NK cells can restrain tumor growth in both liquid and solid settings; however, important lineage-specific differences from CAR-T cells shape design choices. The addition of co-stimulatory end domains such as CD28 and 4-1BB was a defining step for clinically effective CAR-T cells, yet seminal studies indicate that CD28 does not provide analogous co-stimulation in human NK cells (49, 50). In manufacturing, 4-1BB ligand is frequently displayed on “engineered feeder cells” to expand NK cells, consistent with 4-1BB expression on human NK cells (27). Even so, both CD28- and 4-1BB-based end domains have been incorporated into CARs expressed in NK cells, and their respective contributions to NK-cell activation, metabolic fitness, and persistence remain incompletely resolved (51). Unlike *ex vivo*–expanded CAR-T cells, human CAR-NK cells typically exhibit a relatively short lifespan in immunodeficient murine hosts, reinforcing the observation that canonical T-cell co-stimulatory modules do not play the same central role in NK biology. By contrast, vectorized interleukin-15 (IL-15) has emerged as a linchpin for CAR-NK product performance: enforced IL-15 expression markedly augments proliferative capacity and *in vivo* persistence, underscoring its essential role in sustaining long-term NK-cell survival and antitumor activity (52, 53) (**Figure 1**).

Nevertheless, it is important to acknowledge the translational gap between murine models and human outcomes. Standard immunodeficient xenografts often lack the relevant human cytokines and complex TME architecture required to accurately predict CAR-NK persistence. To address this, the field is moving toward humanized mouse models (e.g., MISTRG mice) that support human myeloid and NK cell engraftment, and 3D organoid systems that better recapitulate the physical and immunosuppressive barriers of solid tumors (54). Regarding safety, rigorous preclinical evaluation now extends beyond simple killing assays to include single-cell RNA sequencing (scRNA-seq) to detect off-target activation signatures and tissue cross-reactivity assays using diverse panels of healthy human organs to screen for on-target/off-tumor toxicity before Phase I initiation.

### Lessons from clinical studies with CAR NK cells

Since 2017, more than twenty clinical studies of off-the-shelf, allogeneic CAR-NK therapies have entered the clinic. A landmark phase 1/2 trial evaluated umbilical cord blood (UCB)–derived NK cells engineered with a CD19-specific CAR incorporating a CD28 co-stimulatory endodomain and transgenic interleukin-15 (IL-15), plus an inducible caspase-9 (iC9) safety switch to enable on-demand ablation in the event of toxicity (NCT03056339) (55–57). Following standard lymphodepletion, non-cryopreserved products were infused in a dose-escalation schema and demonstrated a favorable tolerability profile without graft-versus-host disease (GvHD), objective clinical activity in 73% of patients, and *in vivo* expansion with persistence for at least 12 months (58). In an expanded single-center experience (n=37), the platform showed a 1-year progression-free survival of 32%, supporting clinical feasibility while underscoring the need for multicenter validation (56). Notably, the first nine recipients received partially HLA-matched UCB CAR-NK cells, whereas subsequent patients received fully mismatched products; despite complete HLA disparity, CAR-NK persistence approaching 12 months was observed (33, 56, 59). This was unexpected given prior reports that allogeneic, peripheral-blood NK infusions are typically rejected within 2–3 weeks after lymphodepletion, and it highlights a mechanistic question—potentially involving transgenic IL-15 support, product composition, or host milieu—that warrants dedicated investigation (56).

Insights into durability are beginning to emerge. In CAR-T experience, enrichment for central memory and T-stem cell memory phenotypes correlates with expansion and long-term persistence (60–62). While NK-cell memory remains less well defined in humans, murine studies suggest antigen-experienced memory-like features can arise, and human NK cells can acquire a cytokine-induced memory-like (CIML) state after brief exposure to IL-12, IL-15, and IL-18 (63–66). It is therefore plausible that vectorized IL-15 within CAR-NK constructs contributes to sustained *in vivo* fitness and persistence, consistent with observations from the UCB-derived platform (67, 68). Additional donor- and product-level parameters—such as UCB cellular composition, time from collection to preservation, and killer immunoglobulin-like receptor (KIR) mismatch between donor and recipient—have been associated with outcome in early reports but require prospective validation and may not extrapolate to CAR-NK products generated from healthy-donor peripheral blood (41, 69).

Beyond this foundational program, multiple early-phase trials are broadening the evidence base in hematologic malignancies. In B-cell lymphomas, an allogeneic CD19 CAR-NK product expressing membrane-bound IL-15 showed no dose-limiting toxicities and achieved an 80% overall response rate at the highest dose level (NCT05020678) (70). Myeloid leukemias are likewise an active arena: NKG2D-ligand–redirected, membrane-bound IL-15 CAR-NK cells produced a 67% complete response rate in relapsed/refractory AML (NCT04623944), and a separate phase 1 study of CD33-targeted CAR-NK cells reported a favorable safety profile—with cytokine release syndrome in only 1 of 10 patients—and molecular remission in 60% of treated individuals (NCT05008575) (71, 72). While encouraging, these signals still require confirmation in larger, controlled trials to establish reproducibility and durability.

Clinical feedback loops have been instrumental in optimizing delivery regimens. Early trials confirmed that lymphodepletion is not merely to create space but is critical to induce a surge of homeostatic cytokines (IL-15, IL-7) that supports CAR-NK expansion (73). Moreover, the dose-response relationships observed have led to multi-dose strategies rather than single bolus injections to counteract the limited persistence of non-engineered NK cells. Safety controls have also evolved; while GvHD has not been a major issue, the incorporation of inducible suicide switches remains a standard safeguard in iPSC-derived and cord-blood platforms to ensure rapid elimination in case of unexpected severe toxicity.

Parallel advances with induced pluripotent stem cell (iPSC)–derived NK products are beginning to de-risk scalability and standardization. FT576 is an induced pluripotent stem cell-derived natural killer (iPSC-NK) cell therapy engineered to target BCMA (74). Its multi-faceted design incorporates a CAR for direct targeting, a noncleavable Fcγ receptor to potentiate antibody-dependent cellular cytotoxicity, and an IL-15 cytokine-receptor fusion to support cell survival (74). Additionally, the CD38 gene has been deleted to mitigate fratricide (self-targeting) and enable combination therapy with the anti-CD38 monoclonal antibody, daratumumab (71, 74). In a phase 1 multiple myeloma study, no treatment-related adverse events were observed (75). FT596 is an iPSC-derived NK-cell therapy engineered with a CD19-targeted CAR and the same IL-15 receptor–fusion “armoring.” In a phase 1 study for relapsed/refractory B-cell lymphomas (NCT04245722), patients received lymphodepletion followed by FT596, with or without rituximab. The regimen was well tolerated—no grade 3 cytokine release syndrome or neurotoxicity—and showed clinical activity, including a 37% complete response rate (76). Collectively, these early experiences suggest that allogeneic CAR-NK therapy—whether UCB- or iPSC-derived—can couple a strong safety signal with meaningful antitumor activity, while ongoing studies will clarify how best to optimize persistence, standardize manufacturing, and scale access across indications.

## CAR NKT cells

Human natural killer T (NKT) cells are innate-like T lymphocytes that comprise approximately 0.01–0.5% of circulating T cells and are broadly divided into type I and type II subsets (77). This section focuses on type I NKT (invariant NKT, iNKT) cells, which in humans express a highly restricted TCR composed of the invariant Vα24–Jα18 α-chain paired with a limited Vβ11 repertoire (77, 78). iNKT cells physiologically recognize microbial and self-derived lipids presented by CD1d—a monomorphic, MHC-like antigen-presenting molecule expressed on B cells, dendritic cells, and macrophages—thereby enabling donor-unrestricted recognition across individuals (79). Ligation of the invariant TCR elicits rapid effector responses, including robust secretion of interferon-γ (IFN-γ) and other inflammatory mediators (80).

Phenotypically and functionally, NKT cells exhibit a differentiation hierarchy analogous to conventional T cells. In peripheral blood, the majority are CD4 single-positive, with smaller fractions that are CD8 single-positive or double-negative (81). Many NKT cells coexpress canonical NK receptors—such as NKG2D, CD161, and members of the killer cell lectin-like receptor family—which can potentiate iTCR signaling or activate NKT cells independently of TCR engagement, thereby broadening their activation landscape (82–84). In human tumors, NKT-cell infiltration has been documented and is associated with improved survival across several cancer types (85). Mechanistically, NKT cells can directly target CD1d+ malignant cells and eliminate protumorigenic macrophages, reshaping immunosuppressive niches within the tumor microenvironment (86, 87).

Critically, because antigen recognition by NKT cells is CD1d-restricted rather than MHC-restricted, their intrinsic alloreactive potential is minimal; correspondingly, they are associated with a reduced risk of graft-versus-host disease (GvHD) in the setting of allogeneic hematopoietic stem-cell transplantation (84). These properties—donor-unrestricted lipid antigen recognition, capacity to reprogram the myeloid-rich tumor milieu, and low GvHD liability—make type I NKT cells an attractive chassis for chimeric antigen receptor engineering, particularly for allogeneic, off-the-shelf applications.

### Manufacturing of CAR NKT cells

CAR iNKT-cell therapies are being advanced in both autologous and allogeneic formats. Clinical manufacturing typically begins from peripheral blood, followed by immunomagnetic or flow-cytometric enrichment of iNKT cells using the 6B11 antibody, which recognizes the invariant Vα24–Jα18 TCR (iTCR) (84). For *ex vivo* expansion, iNKT cells are stimulated with professional antigen-presenting cells—classically dendritic cells —or, more commonly, with α-galactosylceramide (α-GalCer)–loaded mononuclear feeder cells in the presence of cytokines such as interleukin-2 (IL-2) (88, 89). α-GalCer, a sponge-derived glycolipid presented by CD1d, potently activates iNKT cells and sustains their proliferation in both human and murine systems (84). After robust expansion, the cells are genetically modified (most often with viral vectors) to express a chimeric antigen receptor, yielding CAR iNKT products that retain canonical iNKT functions while gaining antigen-specific targeting (47). These products can be reproducibly cryopreserved with preservation of key phenotypic and functional attributes, enabling lot release, storage, and on-demand clinical use.

Beyond direct isolation from peripheral blood, CAR iNKT cells have also been generated via *ex vivo* differentiation from hematopoietic stem/progenitor cells, producing effectors with cytolytic activity *in vitro* and antitumor efficacy in immunodeficient mouse models (90). A stem cell–based approach enables more uniform, scalable production and may support banked, off-the-shelf allogeneic iNKT therapies by exploiting iNKT cells’ donor-independent, CD1d-restricted recognition to reduce alloreactivity. Concurrently, modern CMC practices tighten process control and harmonize release testing—covering identity, purity, viability, sterility/mycoplasma, endotoxin, vector copy number, CAR expression, and potency measures (e.g., degranulation or target-cell killing)—to deliver consistent product quality across lots and manufacturing sites. Together, these advances position CAR iNKT cells as a practical platform for off-the-shelf and autologous applications alike, with manufacturing flows that integrate well with established clinical cell-therapy logistics.

### Preclinical studies with CAR NKT cells

Human CAR iNKT cells have demonstrated potent antitumor activity *in vivo*, eradicating tumor cells in a preclinical neuroblastoma model in immunodeficient mice, while retaining their physiological iTCR specificity for human CD1d-presented lipids *in vitro* (90). Importantly, these effectors did not precipitate GvHD, indicating that robust antitumor function can be achieved without alloreactive toxicity (91).

As with NK cells, the optimal co-stimulatory architecture for human iNKT cells remains unsettled. CAR designs bearing CD28, 4-1BB, or tandem CD28/4-1BB end domains have been evaluated, yet they generally yielded only modest persistence in immunodeficient mouse models—suggesting that conventional T-cell co-stimulation may be suboptimal for innate-like lineages (92, 93). In sharp contrast, vectorized IL-15 within the CAR cassette consistently augments iNKT proliferation, persistence, and antitumor efficacy without provoking GvHD (94, 95). Beyond proliferative support, IL-15 also confers resistance to hypoxia-associated dysfunction in the TME, thereby sustaining effector competence under metabolic stress.

Insights from murine NKT biology describe transcription factor–defined subsets (NKT1, NKT2, NKT17 characterized by T-BET, GATA3, and RORγt, respectively); analogous stratification is less clearly demarcated in humans and circulating human NKT cells do not sort neatly into naïve, memory, and effector compartments (96). Nevertheless, emerging human data point to a differentiation hierarchy in which L-selectin (CD62L) expression marks an iNKT subset with superior proliferative capacity and persistence potential (97). Cytokine programming further shapes these properties: brief exposure to IL-12 or IL-21 can polarize human iNKT cells toward memory-like states characterized by high CD62L, increased expansion, and durable function (79, 97). Notably, enforced IL-12 expression in human iNKT cells drives a Th1-skewed, memory-like CAR iNKT phenotype that persists long term in immunodeficient mice without inducing GvHD (97).

**Figure 2:**
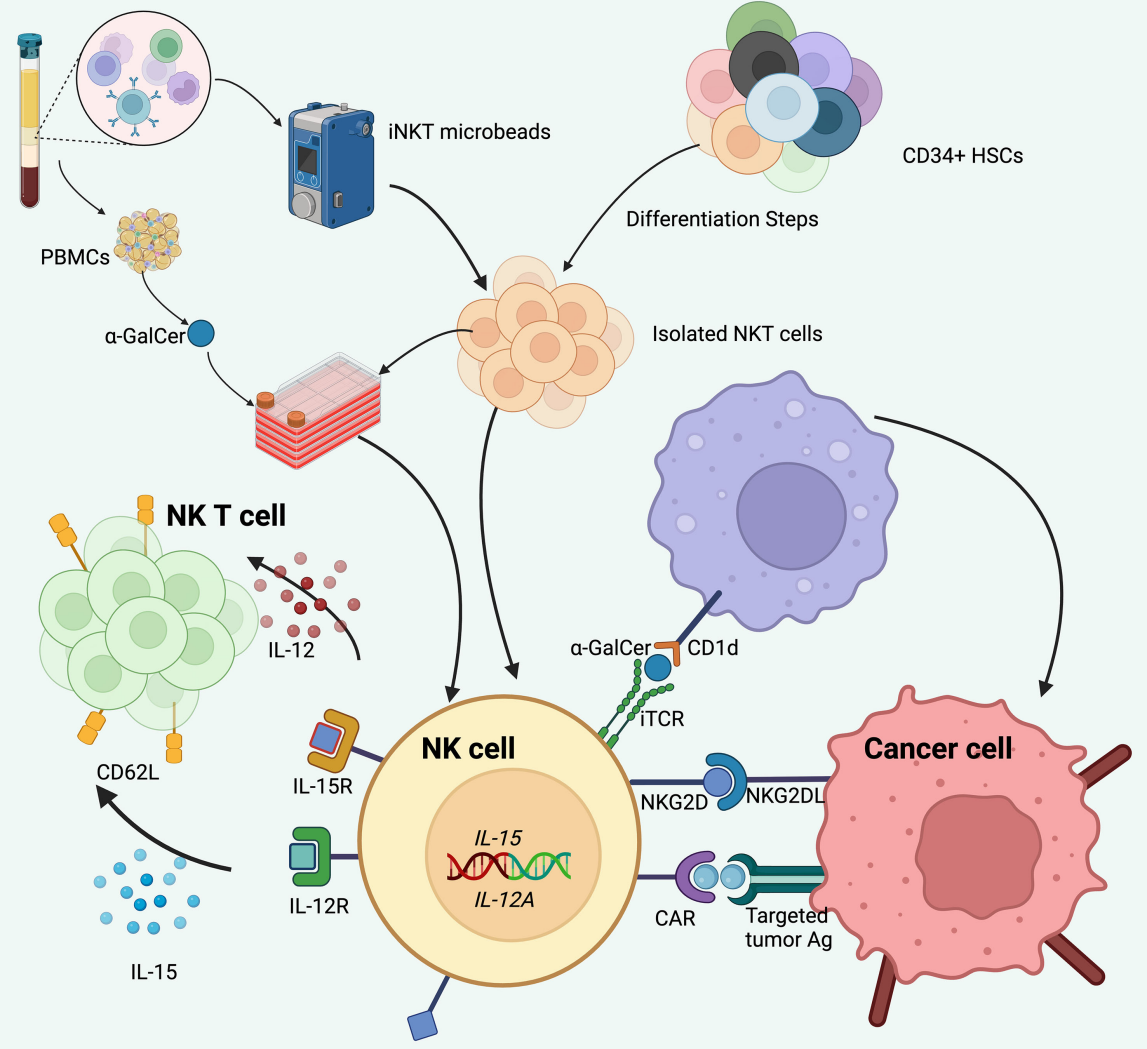
CAR-redirected NKT cells. Human NKT cells can be isolated from peripheral blood through magnetic selection based on their invariant T cell receptor (iTCR) expression and subsequently expanded using autologous mononuclear cell feeders pulsed with α-GalCer in the presence of IL-2. CAR NKT cells may also be produced through multistep differentiation of CD34^+^ hematopoietic stem and progenitor cells (HSPCs). Incorporation of cytokines such as IL-15 or IL-12 into the CAR design plays a critical role in promoting the *in vivo* proliferation, persistence, and antitumor functionality of CAR NKT cells.

Given their CD1d-restricted, donor-unrestricted recognition and minimal alloreactive liability, iNKT cells represent a compelling chassis for banked, allogeneic products. Consistent with this premise, allogeneic CAR iNKT cells have controlled tumor growth in a syngeneic B-cell lymphoma model, underscoring their potential as off-the-shelf therapeutics (98) (**Figure 2**).

### Lessons from clinical studies with CAR NKT cells

A pivotal first-in-human phase 1–2 study evaluated autologous, GD2-targeted CAR iNKT cells in children with relapsed or refractory neuroblastoma (NCT03294954) (99, 100). The product incorporated a CD28 co-stimulatory endodomain and transgenic IL-15 within the CAR cassette. Following lymphodepletion, cryopreserved cells were administered to 12 pediatric participants in a dose-escalation design (47). Treatment was well tolerated, with no dose-limiting toxicities and only manageable cytokine release syndrome (CRS) and yielded an objective response rate of 25% (47). Despite the modest sample size, correlative analyses pointed to IL-15–driven *in vivo* expansion of iNKT cells as a key determinant of clinical activity (47). Moreover, product attributes predicted pharmacodynamics: higher expression of L-selectin (CD62L)—a marker of less-differentiated iNKT subsets—within the infused product correlated with superior expansion and persistence in patients, underscoring the importance of manufacturing strategies that enrich for less-differentiated phenotypes (47).

Building on these autologous data, allogeneic CAR iNKT programs are now in clinical testing for B-cell malignancies (NCT03774654; NCT05487651) (101, 102). In the ANCHOR trial (NCT03774654), patients with relapsed or refractory B-cell cancers receive allogeneic CD19 CAR iNKT cells engineered to co-express IL-15 and edited to delete β2-microglobulin (B2M) and CD74—genetic changes intended to reduce host recognition via MHC class I and II pathways and thereby mitigate rejection (103). Among the first nine treated patients, no treatment-related adverse events were reported, and the overall response rate was 44% including three complete responses, providing an initial proof of concept that banked, off-the-shelf CAR iNKT cells can be delivered safely with encouraging antitumor activity (104). Together, these studies position iNKT cells as a clinically tractable chassis for CAR engineering, while highlighting two levers—IL-15–supported *in vivo* fitness and product composition favoring CD62L^high^, less-differentiated subsets—that are likely to be pivotal for durable responses.

## CAR γδ T cells

Human γδ T cells are a distinct, innate-like T-cell lineage defined by a T-cell receptor (TCR) composed of γ and δ chains and the capacity to recognize antigens without classical MHC restriction (105, 106). Two principal subsets are described: Vγ9Vδ2^+^ cells—which comprise ~50–90% of circulating γδ T cells and ~1–10% of peripheral blood mononuclear cells—sense phosphorylated metabolites (“phosphoantigens”) generated in the isoprenoid biosynthetic pathway (106); and Vδ1+ cells—which are less abundant in blood—engage a broader, less fully defined antigenic space that includes stress-induced ligands (for example, ULBP3, MICA, MICB) and, akin to NKT cells, lipids presented by CD1d (107). Functionally, γδ T cells mount rapid effector responses characteristic of a pre-activated state, releasing perforin and granzymes and producing inflammatory cytokines upon activation (108, 109). Transcriptomic profiling underscores their hybrid identity: γδ T cells share gene programs with both NK cells and conventional αβ T cells, mirroring their role at the interface of innate and adaptive immunity. Consistent with this dual wiring, they express NK receptors (for example, NKG2D and related families) that recognize stress ligands and can either amplify γδ-TCR signaling or trigger TCR-independent activation (110).

In human cancers, γδ T cells infiltrate tumor tissues, and their presence correlates with favorable prognosis across several malignancies (111, 112). They exert direct cytotoxicity against transformed targets (113); tumor-derived phosphoantigens are potent triggers for Vγ9Vδ2^+^ cells, whereas Vδ1^+^ cells can eliminate leukemic populations that resist Vγ9Vδ2^+^ effectors, indicating complementary mechanisms between subsets (114, 115). Crucially, γδ T cells recognize targets in an MHC-independent fashion and exhibit minimal alloreactive potential, properties that align naturally with the development of banked, off-the-shelf allogeneic products. Taken together, the unique antigen-sensing biology, rapid effector capacity, and low risk of graft-versus-host disease position human γδ T cells as a compelling chassis for chimeric antigen receptor (CAR) engineering and clinical translation (116).

### Manufacturing of CAR γδ T cells

Robust clinical protocols now exist to expand human γδ T cells from peripheral blood. Amino bisphosphonates such as zoledronate or pamidronate inhibit farnesyl pyrophosphate synthase in monocytes, driving accumulation of mevalonate-pathway intermediates—most notably isopentenyl pyrophosphate—that selectively activate Vγ9Vδ2^+^ T cells (117). Accordingly, peripheral blood mononuclear cells can be co-cultured with IL-2 and zoledronate to achieve reliable ex vivo expansion of the Vγ9Vδ2^+ subset (118). Autologous, ex vivo–expanded Vγ9Vδ2^+^ T cells have been administered safely to patients with solid tumors, with signals of clinical activity reported across early studies (119). These expanded cells are also amenable to genetic modification: Vγ9Vδ2^+^ products engineered to express CARs have been generated successfully (120). Although manufacturing Vδ1^+^ T-cell products has historically been more challenging, agonistic antibodies that selectively activate and expand human Vδ1^+^ cells have enabled the production of CAR-engineered Vδ1^+^ candidates for prospective clinical use (121, 122).

The therapeutic potency of CAR-γδ-T cells is underpinned by the distinct, yet complementary, surveillance mechanisms of their major subsets (123). Vγ9Vδ2+ cells serve as potent sensors of metabolic dysregulation; they detect the accumulation of phosphoantigens driven by the mevalonate pathway in highly metabolically active tumors (124). This makes them particularly effective against aggressive, high-turnover malignancies but dependent on specific metabolic signatures. In contrast, Vδ1+ cells exhibit a tissue-resident biology with a broader recognition repertoire, targeting stress-induced ligands (MICA/MICB) and lipid antigens presented by CD1d, akin to iNKT cells (125). Their independence from phosphoantigen sensing allows Vδ1+ effectors to target leukemic stem cells or solid tumor clones that may evade Vγ9Vδ2+ recognition. Consequently, the strategic selection or combination of these subsets offers a route to tackle intratumoral heterogeneity: deploying Vγ9Vδ2+ cells to target metabolic hyperactivity while leveraging Vδ1+ cells for tissue-resident surveillance and broad stress-ligand targeting.

### Preclinical studies with CAR γδ T cells

Across multiple models, human CAR γδ T cells secrete pro-inflammatory cytokines upon CAR engagement and mediate antigen-specific antitumor activity *in vitro* and *in vivo* in immunodeficient mice (120, 126). The pharmacologic expansion of Vγ9Vδ2^+^ T cells with zoledronate, coupled with the agent’s established effects in bone metastases, provides a mechanistic rationale to pair CAR Vγ9Vδ2^+^ cells with zoledronate in tumors that seed bone (127). For example, Vγ9Vδ2^+^ CAR T cells directed against a prostate cancer stem-cell antigen have been combined with zoledronate to control osseous disease in preclinical models (128). In parallel, Vδ1^+^ T cells have been engineered with CARs and evaluated preclinically (129, 130); comparative experiments suggest that ex vivo–expanded Vδ1^+^ cells can display superior cytolytic activity relative to Vγ9Vδ2^+^ cells, potentially reflecting their recognition of stress-associated ligands commonly expressed by tumor cells (121, 131).

Co-stimulatory wiring in γδ T cells is only partly mapped. Human γδ T cells express CD28, whose ligation augments IL-2 production, and they possess a functional 4-1BB pathway that promotes proliferation and cytokine release upon engagement (132, 133). These observations justify testing CD28 and 4-1BB end domains in CAR γδ constructs; however, a systematic dissection of how these modules influence γδ T-cell activation, metabolism, persistence, and exhaustion—analogous to their roles in αβ CAR-T cells—remains incomplete (134, 135). Several cytokines (IL-2, IL-12, IL-15, IL-18) are linked to IFN-γ production in human γδ T cells, yet their optimal and safe use to sustain expansion and persistence after adoptive transfer has not been established (136, 137). Notably, constitutive co-expression of IL-15 with a glypican-3 CAR conferred greater intrinsic antitumor activity on human Vδ1^+^ cells in a hepatocellular carcinoma model—without inducing GvHD—highlighting the broadly beneficial role of IL-15 across innate-like platforms (11, 138).

### Clinical experience and lessons learned

CAR γδ T cells are advancing in both autologous and off-the-shelf allogeneic formats. In a Phase 1 trial of allogeneic, CD20-targeted CAR γδ T cells for relapsed/refractory B-cell lymphomas (NCT04735471), lymphodepletion followed by dose-escalated infusion was well tolerated, and adverse events—including cytokine release syndrome and neurologic symptoms—were manageable, with no graft-versus-host disease observed. Of the six evaluable patients, four achieved a complete remission (139, 140). An ongoing phase 1 study (NCT06193486) is testing autologous CAR γδ T cells directed against a prostate cancer stem-cell antigen in men with metastatic disease; per protocol, patients receive zoledronate prior to lymphodepletion and infusion to control bone metastases and potentiate the activity of the transferred cells (141). Although clinical datasets remain limited, prior trials using non-engineered γδ T cells have established feasibility and a favorable safety profile—importantly, without GvHD in allogeneic settings (142). Still, definitive evidence of robust *in vivo* expansion and long-term persistence for either autologous or allogeneic CAR γδ products is not yet available. Early remissions in B-cell malignancies suggest that rational enhancements—such as cytokine armoring—may further amplify efficacy, and next-generation trials are poised to test these hypotheses prospectively (143).

## CAR macrophages

Macrophages are ubiquitous, tissue-resident phagocytes that arise largely from circulating monocytes and serve essential homeostatic functions across organs, including the clearance of senescent and apoptotic cells and the coordination of tissue repair following inflammatory injury (144). Although highly plastic and responsive to diverse microenvironmental cues, a clinically useful shorthand frames their polarization along an M1–M2 continuum (145). “Classical” M1-like macrophages, primed by interferon-γ (IFN-γ) and microbial products such as lipopolysaccharide, mount microbe- and tumor-directed responses characterized by robust secretion of pro-inflammatory cytokines (IL-1β, IL-6, TNF) and high output of inducible nitric oxide synthase (iNOS) and reactive oxygen species (146). In contrast, “classical” M2-like macrophages are induced by IL-4 or IL-13, emphasize tissue remodeling and the dampening of inflammation to limit collateral damage, express scavenger receptors including the mannose receptor, and release anti-inflammatory mediators such as IL-10 (146, 147).

Within tumors, macrophages are abundant infiltrates TAMs that often comprise mixtures of M1-like, anti-tumor effectors and M2-like, pro-tumor populations (148, 149). This coexistence poses a central dilemma for cell therapy: M2-skewed TAMs can blunt CAR-T efficacy by suppressing T-cell activation, impairing trafficking, and fostering angiogenesis and matrix remodeling, whereas M1-like TAMs can enhance antigen presentation, produce IL-12–rich inflammatory tone, and support lymphocyte infiltration. Consequently, two complementary engineering strategies have emerged. One seeks to equip CAR-T cells to selectively deplete or reprogram M2-like macrophages—via targeted killing or by delivering re-polarizing signals—thereby relieving myeloid-mediated immunosuppression (87, 149, 150). The other directly harnesses macrophages as effectors: CAR-macrophages (CAR-M) are designed to augment Fc- and CAR-triggered phagocytosis, amplify production of reactive oxygen/nitrogen species, and convert intratumorally antigen capture into T-cell priming through antigen presentation, offering both cytoreductive and immuno-educative benefits (151–153). Together, these approaches exploit macrophage plasticity: either tilt resident TAMs toward an M1-dominant, therapy-permissive state or deploy engineered macrophages to attack tumor cells and remodel the tumor microenvironment in favor of durable antitumor immunity.

### Manufacturing of CAR macrophages

Clinical-grade CAR-macrophage (CAR-M) production is still in an early translational phase. A common workflow isolates CD14^+^ monocytes from peripheral blood and differentiates them ex vivo into macrophages using granulocyte–macrophage colony-stimulating factor (GM-CSF) (154). Compared with lymphocytes, stable gene delivery into monocytes/macrophages is less straightforward, owing in part to their antiviral sensing and transduction resistance. Nonetheless, macrophages are amenable to genetic modification with adenoviral vectors—particularly Ad5f35—which exploit CD46, a surface receptor broadly expressed on human monocytes/macrophages and used by several pathogens, including adenoviruses (155). Following engineering and expansion, CAR-M products intended for clinical use can be cryopreserved with retention of identity and core functions after thaw, enabling batch release and on-demand administration.

To improve standardization and scalability, alternative starting materials are being evaluated. These include macrophages derived from the well-characterized monocytic tumor cell line THP-1 and induced pluripotent stem cells (iPSCs) (156). Line- or iPSC-based routes offer practical advantages—clonal banking, uniform phenotype, and reproducible yields—and can simplify chemistry, manufacturing, and controls by anchoring processes to qualified master cell banks (156). Across programs, current lot-release testing typically includes identity (e.g., CD68 and CSF1R expression), purity and viability, sterility and endotoxin, vector-related attributes where applicable, and potency assays—such as phagocytosis of opsonized targets, antigen-specific cytotoxicity, and cytokine production. As these platforms mature, standardizing differentiation protocols, vector systems, and cryopreservation specifications will be critical to achieving scalable, reproducible manufacture of CAR-M products while preserving the lineage-specific advantages that motivate their development (157).

### Preclinical studies with CAR macrophages

CAR-M have been shown to recognize tumor-associated antigens on malignant cells and to engulf those targets through antigen-specific phagocytosis (158). Compared with CAR-T cells, CAR-M may enjoy a trafficking advantage in desmoplastic, stroma-rich tumors: macrophages natively infiltrate and remodel tissue by secreting matrix-degrading metalloproteinases, potentially improving penetration into the TME and access to cancer cells entrenched in dense extracellular matrix (159). Beyond direct tumor clearance, CAR-M can process engulfed antigens and may secondarily prime adaptive immunity, offering a route to both immediate cytoreduction and immunologic “education” of the TME (160).

Preclinical work also underscores that CAR designs optimized for lymphocytes are unlikely to be plug-and-play in macrophages. While the antigen-binding domain (for example, an scFv) remains essential for target recognition, the optimal intracellular wiring is distinct: canonical CD3ζ ITAM signaling that efficiently triggers cytotoxic degranulation in T cells appears suboptimal for professional phagocytes, whereas modules that emulate Fc receptor common γ-chain signaling may better drive opsonophagocytic programs (161). Along the same lines, embedding Toll-like receptor 4 (TLR4)–derived signals within the CAR can bias macrophages toward an M1-like, pro-inflammatory state, potentially enhancing antitumor activity and T-cell support (162). Complementary engineering levers are being explored—such as phagocytic receptor motifs (e.g., FcRγ, MerTK, MEGF10), SIRPα–CD47 axis interference, and chemokine-receptor tuning—to align CAR-M activation with engulfment, antigen presentation, and productive TME remodeling (163).

Reprogramming macrophages for CAR therapy introduces a unique homeostatic challenge: maintaining a pro-inflammatory M1-like phenotype within a tumor microenvironment that strongly drives macrophages toward an M2-like, immunoregulatory state (164, 165). Unlike T cells, whose primary function is cytotoxicity, macrophages must resist re-education by tumor-derived cytokines such as IL-4 and IL-10, which can diminish their antitumor functions and promote tissue-remodeling phenotypes (166). To overcome this, emerging CAR-M engineering strategies attempt to strengthen macrophage-intrinsic activation programs and decouple antigen recognition from conventional phagocytic pathways. For example, intracellular signaling domains derived from FcRγ or MEGF10 have been incorporated into CAR constructs to enhance antibody-like opsonophagocytic activity, in some cases outperforming canonical CD3ζ-based designs in macrophage systems (167). In parallel, approaches to stabilize M1 polarization—such as augmenting TLR4-driven NF-κB signaling or modulating metabolic checkpoints including ACOD1/Irg1, which governs itaconate production—have demonstrated improved resistance to M2-skewing cues in preclinical models (168, 169). Collectively, these engineering strategies aim to convert macrophages from passive scavengers into robust, inflammation-competent effector cells capable of sustaining antitumor immunity and coordinating downstream T-cell recruitment.

Unbiased CRISPR–Cas9 screening has begun to reveal macrophage-intrinsic metabolic checkpoints of phagocytosis; for example, deletion of aconitate decarboxylase 1 (ACOD1/IRG1) significantly increased CAR-M phagocytic capacity (168, 170). These findings suggest that coupling CAR signaling with targeted metabolic rewiring could further potentiate efficacy. At the same time, key safety questions remain unresolved: whether highly activated CAR-M could provoke excessive inflammation, off-tumor phagocytosis, or bystander tissue injury in clinical settings is not yet known. Incorporating controllable safety devices (e.g., suicide switches), tumor-restricted promoters, and dose-finding strategies—together with rigorous biodistribution and cytokine-profiling studies—will be essential as the field advances CAR-M toward first-in-human applications.

### Lessons from clinical studies with CAR macrophages

Encouraging preclinical efficacy of HER2-directed CAR-M prompted a first-in-human, phase 1 trial in HER2-positive solid tumors (NCT04660929) (171). In this study, autologous products were generated by differentiating peripheral blood monocytes into M1-polarized macrophages ex vivo and transducing them with a HER2-specific CAR using an Ad5f35 vector, followed by intravenous infusion on a dose-escalation schema (171). Unlike most CAR-T protocols, patients did not receive lymphodepleting preconditioning—reflecting the expectation that macrophages would not depend on the homeostatic cytokine surge (for example, IL-7/IL-15) that supports CAR-T expansion and persistence. Recently reported data from 14 participants with relapsed/refractory breast or gastroesophageal cancers showed a favorable safety profile without dose-limiting toxicities; however, antitumor activity was limited, with only transient disease stabilization observed in 4 of 8 treated patients (172).

These results underscore two immediate development priorities: optimizing potency/persistence and addressing vector-related immunogenicity. Because adenoviral engineering may render CAR-M susceptible to rapid immune clearance, alternative strategies are being explored. Demonstrating the safety of intravenously administered CAR-M opens the door to next-generation approaches, including (i) CAR-modified monocytes that differentiate into macrophages *in vivo* to enhance tissue seeding and durability, and (ii) direct *in vivo* reprogramming of resident macrophages using improved gene-delivery platforms—such as modified viral vectors or lipid nanoparticles—that could reduce manufacturing complexity and potentially improve biodistribution (173).

## Conclusion

The clinical success of autologous CAR-T cell therapy in hematologic malignancies stands as a watershed moment in oncology, yet it has simultaneously exposed the inherent limitations of the “bespoke” manufacturing model. As we dissect in this review, the reliance on patient-specific supply chains not only imposes prohibitive costs and logistical delays but also restricts the biological potential of the final product, which is often derived from a heavily pre-treated, exhausted immune repertoire1. In this context, the pivot toward innate and innate-like effectors—specifically NK cells, iNKT cells, γδ-T cells, and macrophages—represents not merely an incremental update, but a fundamental paradigm shifts toward truly “off-the-shelf” immunotherapy. The most compelling advantage of these innate lineages is their intrinsic safety profile regarding alloreactivity. Unlike conventional αβ T cells, which require complex gene editing to prevent GvHD, innate effectors utilize MHC-independent recognition mechanisms3. NK cells target “missing-self” and stress ligands; iNKT and γδ-T cells recognize conserved lipid antigens and phosphoantigens, respectively; and macrophages rely on phagocytic signaling. This biology naturally aligns with the industrial goal of creating standardized, bankable drug products that can be administered on demand, thereby democratizing access to cellular therapy. However, translating this promise into durable clinical benefit, particularly for solid tumors, requires overcoming two interconnected “Valleys of Death”: biological persistence and manufacturing consistency. First, regarding persistence, early clinical trials have highlighted that innate cells, while safe, are prone to rapid exhaustion and clearance in the absence of homeostatic cytokine support. The “first wave” of engineering solutions—primarily the incorporation of constitutive IL-15 signaling—has proven essential for sustaining *in vivo* fitness. Yet, future success will demand more sophisticated strategies. Emerging trends point toward “metabolic armoring,” such as protecting cells from oxidative stress or nutrient deprivation in the TME, and “allo-evasion” engineering to prevent host immune rejection of the transferred cells. The goal is to transform these effectors from transient “killer drugs” into persistent “living drugs” that can maintain surveillance against relapse. Second, the challenge of manufacturing consistency cannot be overstated. The current landscape is fragmented, with varying donor sources and expansion protocols leading to significant batch-to-batch heterogeneity. Here, the transition toward iPSC platforms offers a transformative solution. By enabling the generation of clonal master cell banks that can be precisely edited and validated before differentiation, iPSC technology promises to convert cell therapy manufacturing from a variable “process-based” art into a reproducible “product-based” science. Furthermore, perfecting cryopreservation techniques is critical; “off-the-shelf” availability is meaningless if the thawing process compromises effector viability or cytotoxicity. Looking to the horizon, the next generation of innate CAR therapies will likely move beyond simple direct killing. We envision “lineage-aware” combinatorial strategies that exploit the unique biology of each cell type. For instance, CAR-iNKT cells or CAR-macrophages could be deployed as a “vanguard” to remodel the immunosuppressive stroma and deplete myeloid-derived suppressor cells, paving the way for a subsequent wave of CAR-T or CAR-NK effectors. Additionally, the field is beginning to explore *in vivo* programming, utilizing lipid nanoparticles (LNPs) or viral vectors to transduce macrophages or NK cells directly inside the patient. This approach could theoretically bypass the complexities of ex vivo manufacturing entirely, representing the ultimate evolution of the “off-the-shelf” concept.

In summary, while the path forward is challenged by the complexities of the solid tumor microenvironment and the demands of industrial scaling, the trajectory is clear. By harnessing the diverse, evolutionarily conserved mechanisms of innate immunity and coupling them with precision engineering, we are moving closer to a future where effective, safe, and affordable cell therapies are available to every patient, not as a last resort, but as a standard of care.
